# Early EEG Alterations Correlate with CTP Hypoperfused Volumes and Neurological Deficit: A Wireless EEG Study in Hyper-Acute Ischemic Stroke

**DOI:** 10.1007/s10439-021-02735-w

**Published:** 2021-02-18

**Authors:** Miloš Ajčević, Giovanni Furlanis, Aleksandar Miladinović, Alex Buoite Stella, Paola Caruso, Maja Ukmar, Maria Assunta Cova, Marcello Naccarato, Agostino Accardo, Paolo Manganotti

**Affiliations:** 1 Department of Engineering and Architecture, University of Trieste, Via A. Valerio 10, 34127 Trieste, Italy; 2 Clinical Unit of Neurology, Department of Medicine, Surgery and Health Sciences, Trieste University Hospital ASUGI, University of Trieste, Strada di Fiume 447, 34149 Trieste, Italy; 3 Radiology Unit, Department of Medicine, Surgery and Health Sciences, Trieste University Hospital ASUGI, University of Trieste, Strada di Fiume 447, 34149 Trieste, Italy

**Keywords:** EEG, CT perfusion, Hyperacute ischemic stroke, NIHSS, Biomedical signal processing

## Abstract

Brain electrical activity in acute ischemic stroke is related to the hypoperfusion of cerebral tissue as manifestation of neurovascular coupling. EEG could be applicable for bedside functional monitoring in emergency settings. We aimed to investigate the relation between hyper-acute ischemic stroke EEG changes, measured with bedside wireless-EEG, and hypoperfused core-penumbra CT-perfusion (CTP) volumes. In addition, we investigated the association of EEG and CTP parameters with neurological deficit measured by NIHSS. We analyzed and processed EEG, CTP and clinical data of 31 anterior acute ischemic stroke patients registered within 4.5 h from symptom onset. Delta/alpha ratio (DAR), (delta + theta)/(alpha + beta) ratio (DTABR) and relative delta power correlated directly (*ρ* = 0.72; 0.63; 0.65, respectively), while alpha correlated inversely (*ρ* = − 0.66) with total hypoperfused volume. DAR, DTBAR and relative delta and alpha parameters also correlated with ischemic core volume (*ρ* = 0.55; 0.50; 0.59; − 0.51, respectively). The same EEG parameters and CTP volumes showed significant relation with NIHSS at admission. The multivariate stepwise regression showed that DAR was the strongest predictor of NIHSS at admission (*p* < 0.001). The results of this study showed that hyper-acute alterations of EEG parameters are highly related to the extent of hypoperfused tissue highlighting the value of quantitative EEG as a possible complementary tool in the evaluation of stroke severity and its potential role in acute ischemic stroke monitoring.

## Introduction

Stroke is associated with immediate brain changes resulting in a decrease of cerebral blood perfusion inducing the reduction of oxygen and glucose supply, leading to cerebral infarction.^4^ Neuroimaging, together with clinical assessment, has been proving to play a key role in ischemic stroke diagnosis and particularly to determine the eligibility of patients for reperfusion therapy.^18^,^20^,^26^,^32^,^44^ The most adopted evaluation method for stroke-related neurologic impairment is the 11-item NIHSS.^1^ MRI- or CT Perfusion- (CTP) techniques can identify the ischemic core and the salvageable hypoperfused penumbra,^33^ while pointing out which patients can best benefit from the reperfusion treatments (thrombolysis and thrombectomy),^20^ also in cases of wake-up stroke.^2^ CTP is increasingly used in hyper-acute stroke assessment due to its distinctively short imaging time ensuring at the same time high sensitivity (80%) and specificity (95%).^32^ MRI- and CT-based perfusion imaging techniques are not feasible tools to monitor brain ischemia evolution in the acute phase.

Encephalogram (EEG) could be applicable for bedside functional monitoring in emergency setting.^3^,^38^,^41^ EEG is a non-invasive technique, characterized by high temporal resolution and it enables fast evaluation of instantaneous brain function. Moreover, it shows good sensitivity to acute changes in cerebral blood flow (CBF) 35,36 and neural metabolism.^30^

Brain oscillatory activity changes occurring in acute ischemic stroke are related to neurophysiological changes in the cerebral tissue during hypoperfusion as manifestation of neurovascular coupling.^34^,^41^ The reduction of CBF in ischemic areas leads to changes in EEG activity, namely increased power especially in delta frequencies and decreased power in alpha frequencies.^23^ EEG alterations during the sub-acute and post-acute phase of ischemic stroke have been widely studied.^13^,^15^,^37^,^47^

Yet, only a few have investigated the earliest (< 4.5 h from symptom onset) EEG alterations. To our best knowledge the relation between EEG spectral parameters and hypoperfused volumes measured by CTP, and neurological deficit at admission has not been studied yet in the hyper-acute stroke phase. We aimed to investigate the relation between stroke-related EEG changes, measured on bedside with wireless EEG, and hypoperfused core/penumbra CTP estimated volumes during the earliest phase of ischemic stroke. In addition, EEG and CTP parameters were correlated with neurological deficit at admission.

## Materials and Methods

### Study Population and Protocol

Thirty-one consecutive patients (mean age 78.5 ± 10.9; 18 female, 13 male) with acute ischemic stroke, admitted to the Stroke Unit of the University Medical Hospital of Trieste (Trieste, Italy), who underwent CTP and EEG recording at the bedside within 4.5 h from known stroke onset were included in this study. Due to lower sensibility of CTP, patients with posterior circulation stroke were excluded. Unknown stroke onset and wake-up stroke and stroke-mimic were excluded. Previous stroke, hematic effusion, history of epileptic seizure, pre-morbid dementia, use of medication such as neuroleptic or benzodiazepines, were also exclusion criteria due to their effect on EEG assessment.

All patients underwent neurological examination at admission including National Institutes of Health Stroke Scale (NIHSS) and a multimodal CT imaging protocol, comprising cerebral non-contrast CT (NCCT), CT angiography (CTA) and CTP. In the timespan between CTP and the decision of possible reperfusion treatment, if the conditions were suitable in the emergency setting, EEG was acquired with a pre-wired headcap and wireless EEG device. After this assessment, if the inclusion criteria were respected, patient underwent to thrombolysis and/or thrombectomy.

In addition, 10 healthy age-matched (mean age 74.3 ± 6.7; 6 female, 4 male) were recruited and underwent EEG recordings in order to compare the EEG parameters with those calculated in hyper-acute stroke. Exclusion criteria was a history of neurologic and psychiatric disorders, such as depressive disorders, anxiety, stroke, brain injury, epilepsy and dementia. Each subject underwent Montreal Cognitive Assessment (MoCA)^24^ test in order to exclude cognitive impairment. No differences in age (*p* = 0.259) and sex (*p* = 0.913) between enrolled stroke patients and healthy control group were detected.

This study was approved by the Local Ethics Committee CEUR (Comitato Etico Unico Regionale, FVG, Italy) with approval number 115/2018. The research was conducted according to the principles of the Declaration of Helsinki. All participants released their informed consent.

### EEG Acquisition and Processing

EEG was acquired at bedside within 4.5 h from stroke symptom onset by using 19 channel 10–20 Ag/AgCl electrodes headset and Be Plus LTM amplifier @64 channels Wi-Fi (EBNeuro, Florence, Italy). The wireless EEG device, characterized by high CMRR and low noise, preamplified, conditioned, and sampled acquired EEG signals immediately near to the electrodes to reduce electromagnetic interference, minimize movement of electrode wires and electrode displacement that can dramatically degrade EEG signal quality. EEG signals were then transmitted *via* Wi-Fi protocol to a base station allowing registration in an emergency setting without interfering with standard patient management procedure. All electrode impedances were kept below 5 kΩ and sampling rate was set to 128 Hz. The off-line analysis was performed by scripts developed in MATLAB (MathWorks Inc., Natick, MA). All signals were digitally filtered with the 0.5–40 Hz 2nd order Butterworth bandpass filter and the first 60 s of the artifact free EEG were analyzed. Power spectral density (PSD) was estimated for each channel using Welch’s periodogram,^45^ averaged on 11 tracts of 10 s each, windowed with a Hann window, with 50% overlap. The relative power for each of spectral bands (delta: 1–4 Hz; theta: 4–8 Hz; alpha: 8–13 Hz; beta: 13–30 Hz) was calculated for each channel. The relative powers were obtained by normalizing with a total power across the 1–30 Hz range. In addition, (delta + theta)/(alpha + beta) ratio (DTABR) and delta/alpha ratio (DAR) were computed. Relative power for each band, DAR and DTABR parameters were averaged over all nineteen scalp electrodes.

### CTP Acquisition and Processing

**Figure 1:**
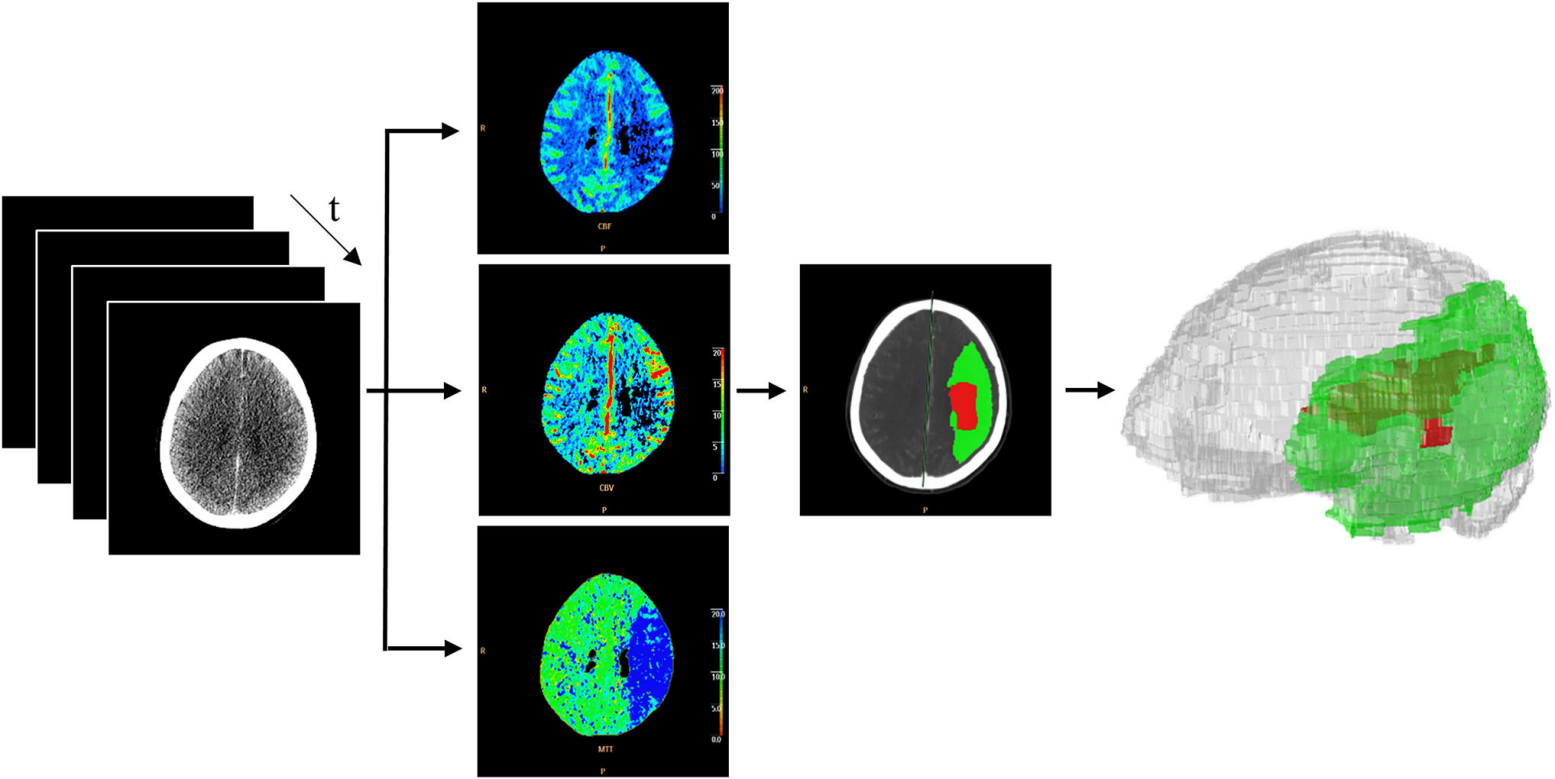
CTP analysis. From left to right: source CTP data; supratentorial CBF, CBV and MTT calculated maps, from top to bottom, respectively; core (red)-penumbra (green) map; 3D representation of total hypoperfused supratentorial volume (core + penumbra).

All CTP scans were acquired on a 256-slice Philips Brilliance iCT scanner (Philips Healthcare, Best, The Netherlands) at 80 kVp and 150–200 mAs. At initiation of scanning, 75 mL of contrast medium was injected intravenously at a rate of 4 mL/s, followed by a 40 mL of saline bolus. The three-dimensional axial acquisitions on a whole brain volume with a reconstruction of the slices set to 5 mm were performed. The acquisitions were carried out every 4 s, resulting in a total scanning time of 60 s. CTP source image processing was performed by Extended Brilliance Workstation v 4.5 (Philips Medical Systems, Best, Netherlands) and in-house developed in Matlab (MathWorks Inc., Natick, MA), as previously described.^2^,^19^ CTP analysis is summarized in Fig. 1. The perfusion maps mean transit time (MTT), cerebral blood volume (CBV) and cerebral blood flow (CBF) were calculated from source CTP. Gaussian curve fitting by least mean squares method was applied to obtain mathematical descriptions of the time-density curves for each voxel. An arterial input function and venous output are selected and subsequently a closed-form deconvolution was applied to produce a MTT map.^7^ CBV map was calculated from the area under the time attenuation curve and finally CBF map as a ratio between CBV and MTT. Ischemic core and penumbra areas were identified by application of specific thresholds,^46^ i.e. MTT voxels > 145% of the contralateral healthy area and CBV < 2.0 mL/100 g, and MTT voxels > 145% of the contralateral healthy area and CBV > 2.0 mL/100 g, respectively. Ischemic core volume and total hypoperfused volume excluding artifacts was calculated by integration of identified voxels as described in a previous study.^19^

### NIHSS Assessment

NIHSS evaluation was carried out at the time of presentation at the Stroke Unit by a vascular neurologist trained in performing NIHSS examination. The NIHSS test was performed within 15 min before CTP scanning. NIHSS consists of 11 items assessing the main neurologic functions, such as eye movement, visual fields, coordination, motor strength, sensation, neglect, and language. NIHSS score ranges from 0 to 42, with 7 points attributed to language functions (2 points for orientation, 2 points for command execution, 3 points for aphasia).

### Statistical Analyses

Variables were presented with mean and standard deviation or median and range depending on the distribution. Kolmogorov–Smirnov test was used to evaluate normal distribution of variables. The Spearman correlation was used to determine the degree of correlation between hypoperfused volumes and the EEG spectral parameters and ratios. A two-sided Wilcoxon rank sum test was used to investigate the differences between EEG parameters calculated in hyper-acute stroke patients and healthy controls. The correlation between NIHSS at admission and EEG and CTP parameters were also investigated. Stepwise multiple regression analysis was subsequently adopted to identify the most relevant independent parameters related to acute neurological disability using NIHSS on admission. A *p* < 0.05 was considered statistically significant.

## Results

Patients’ demographic, clinical and radiological data are summarized in Table 1. The median neurological impairment at admission was NIHSS = 8 (3–24). ASPECT score of 9 (range 6–10) assessed on NCCT was observed. Median time between symptoms onset and EEG recording was 178 min (range 82–261 min). The CTP analysis showed a median total hypoperfused volume, core volume and mismatch of 56.0 (2.0–219.0) mL, 2.5 (0–102.0) mL, 0.92 (0.13–1.0), respectively.

**Table 1:** Participants’ demographics, clinical and radiological characteristics.

Personal characteristics	*n* = 31
Age [years]	78.5 ± 10.9
Sex F/M	18/13
Symptom onset—EEG assessment [min]	178 (82–261)
ASPECTS	9 (6–10)
NIHSS at admission	8 (3-24)
Anamnestic mRS	0 (0–4)
Lesion side of the lesion L/R [*n*]	17/14
Bamford stroke subtypes	
TACI	7 (23%)
PACI	22 (71%)
LACI	2 (6%)
TOAST classification	
Atherothrombotic	6 (19%)
Lacunar	2 (6%)
Cardioembolic	12 (39%)
Cryptogenic	10 (32%)
Other cause	1 (3%)
CTP parameters	
Total hypoperfused tissue [mL]	56.0 (2.0–219.0)
Core [mL]	2.5 (0–102.0)
Mismatch	0.92 (0.13–1.0)
HTN [*n* (%)]	15 (74%)
DM [*n* (%)]	15 (47%)
Dyslipidemia [*n* (%)]	17 (50%)
AF [*n* (%)]	12 (35%)
ICP [*n* (%)]	7 (21%)

Participants’ reported age (years), sex (*n*), symptom onset—EEG assessment [min], ASPECTs, NIHSS at admission, anamnestic mRS, lesion side (*n*), Bamford stroke subtypes (%). TOAST classification (%), CT perfusion parameters (mL), history of hypertension (HTN, %), diabetes (DM, %), dyslipidemia (%), atrial fibrillation (AF, %), ischemic cardiomyopathy (ICM, %)

*TACI* total anterior circulation infarct, *PACI* partial anterior circulation infarct, *LACI* Lacunar stroke

**Figure 2:**
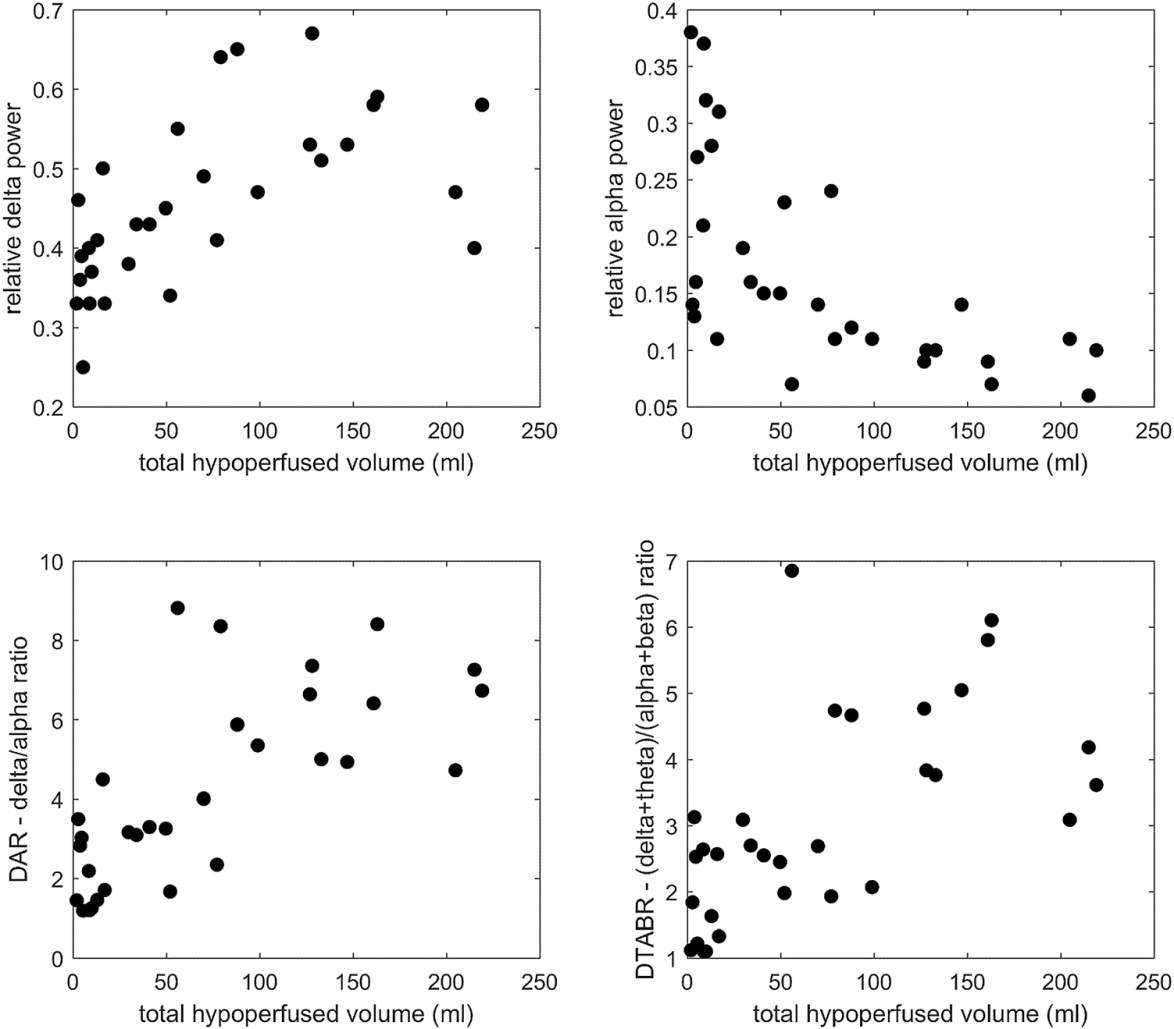
Delta and alpha relative powers, as well as DAR and DTABR ratios were plotted against total hypoperfused volume. Delta relative power, DAR, DTABR showed linear dependency, while for alpha relative power an inverse power law relation was observed.

In Table 2 are reported median (range) values of EEG extracted parameters and their correlation with CTP volumes and neurological deficit at admission. DAR, DTBAR and relative delta power correlated directly, while alpha correlated inversely with total hypoperfused volume as well as with ischemic core. In Fig. 2, delta and alpha relative powers, as well as DAR and DTABR ratios, were plotted against total hypoperfused volume. Delta relative power, DAR, DTABR showed linear dependency, while for alpha relative power an inverse power law relation was observed.

**Table 2:** Correlation between EEG spectral parameters and total hypoperfused volume, ischemic core volume and NIHSS, respectively.

EEG spectral parameter	Median (range)	Spearman’s *ρ* (*p*-value)		
		vs. total hypoperfused volume	vs. core volume	vs. NIHSS
Delta	0.46 (0.25–0.67)	0.65 (*p* < 0.001)	0.59 (*p* = 0.002)	0.78 (*p* < 0.001)
Theta	0.22 (0.09–0.35)	0.05 (*p* = 0.831)	0.06 (*p* = 0.811)	0.03 (*p* = 0.897)
Alpha	0.13 (0.06–0.38)	− 0.66 (*p* < 0.001)	− 0.51 (*p* = 0.012)	− 0.75 (*p* < 0.001)
Beta	0.14 (0.05–0.22)	− 0.05 (*p* = 0.818)	− 0.01 (*p* = 0.970)	− 0.31 (*p* = 0.137)
DAR	3.75 (1.21–8.81)	0.72 (*p* < 0.001)	0.55 (*p* = 0.005)	0.81 (*p* < 0.001)
DTABR	2.67 (1.10–6.85)	0.63 (*p* < 0.001)	0.50 (*p* = 0.013)	0.86 (*p* < 0.001)

**Figure 3:**
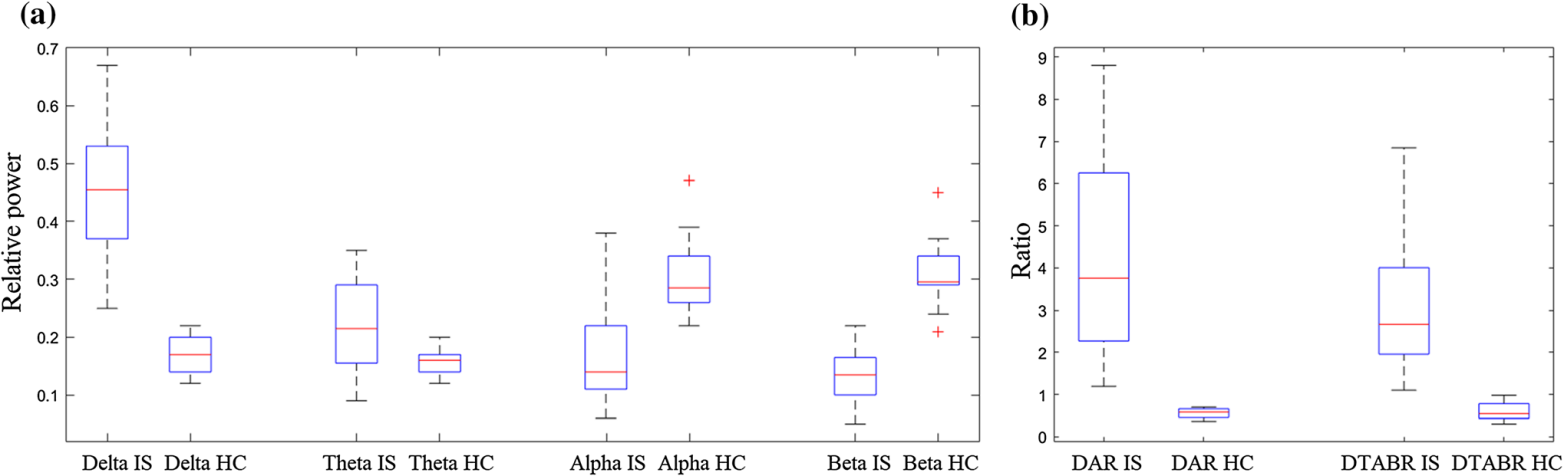
Comparison of EEG parameters extracted in hyper-acute stroke patients and healthy age-matched controls. Box and whisker plot. Panel (**a)** delta, theta, alpha and beta relative powers. Panel (**b)** Delta/alpha-*DAR* and (delta+theta)/(alpha+beta)-*DTABR* ratios. *IS* ischemic stroke, *HC* healthy controls. All EEG parameters differed significantly between hyper-acute ischemic stroke patients and age-matched healthy controls.

Box and whisker plot of EEG parameters extracted in hyper-acute stroke patients and 10 healthy age-matched controls is reported in Fig. 3. Relative delta (*p* < 0.001) and theta (*p* = 0.006) powers were significantly higher in stroke patients compared to healthy subjects, while relative alpha (*p* = 0.002) and beta (*p* < 0.001) powers were significantly lower. In addition, also DAR and DTABR were significantly higher in stroke patients (*p* < 0.001 and *p* < 0.001, respectively).

Regarding, the relation of EEG parameters and neurological deficit at admission, the strong correlation of DAR, DTBAR, delta and alpha with NIHSS was observed (*ρ* = 0.81, *p* < 0.001; *ρ* = 0.86, *p* < 0.001; *ρ* = 0.78, *p* < 0.001; *ρ* = − 0.75, *p* < 0.001, respectively). Furthermore, CTP parameters were related to neurological deficit. A positive significant correlation was found between total hypoperfused volume and neurological deficit (*ρ* = 0.75; *p* < 0.001) as well as between ischemic core and neurological deficit (*ρ* = 0.69; *p* < 0.001). The multivariate stepwise regression showed the DAR was the strongest predictor of NIHSS on admission (beta = 0.828; *p* < 0.001).

## Discussion

The ischemia to infarction transition in the ischemic stroke acute phase is a rapidly developing process (from few minutes to few hours) causing cellular death in hypoperfused brain tissue.^29^ EEG alterations are related to neurophysiological changes in the brain tissue during hypoperfusion, as expression of neurovascular coupling.^41^ The main finding of this study is a significant correlation between stroke-related EEG alterations and the total hypoperfused volume measured by CTP in the ischemic stroke hyper-acute phase (< 4.5 h from symptom onset). In particular, DAR, DTBAR and relative delta power correlated directly, while alpha correlated inversely with total hypoperfused volume as well as with neurological deficit at admission. In addition, the same was observed in moderate manner for ischemic core volume.

This is the first study which investigates the relationship between brain oscillatory activity and CTP volumes, and neurological deficit in the first 4.5 h of hyper-acute ischemic stroke. In the past, a few studies compared EEG oscillatory activity alteration with ischemic area assessed by neuroimaging, yet not in the hyper-acute phase. Abnormal EEG delta waves foci correlated with areas of cerebral lesion determined by NCCT, PET and MRI techniques.^10^,^13^,^29^,^30^ By comparing NCCT, performed at 4 days after stroke’s onset, and EEG, recorded within 24 h from onset, highlighted a relation between the site of increase of delta power and anatomical position of parenchymal damage.^29^ Yet, NCCT is not able to identify morphological changes in the hyper-acute phase of ischemic stroke.^10^ A modest correlation between alteration of delta activity assessed by EEG and 15-h DWI was reported by Finnigan *et al.*^13^

The joint analysis of the EEG and hemodynamic activity assessed by perfusion neuroimaging is gaining increasing interest in the initial phase of ischemic stroke. EEG recording and DWI-PWI MRI sequences allowed direct correlation EEG abnormalities with functional MRI changes in rodent models of ischemic stroke.^28^ A recent study reported agreement between slow rhythms hemispheric prevalence on EEG maps and lesion side assessed using CTP in patients with hyper-acute stroke.^41^

Our results showed that the larger CTP volumes are related to bilateral linear increase of delta, DAR and DTBAR parameters and inverse power law decrease of alpha waves in the early phase. The diffused bilateral EEG changes are consistent with previous findings which report that diffused alterations may reflect the early phase of brain ischemia^38^ and early changes in interhemispheric connectivity.^5^,^43^ In addition, we observed that these EEG spectral parameters were more related to total hypoperfused volume than to infarct core, indicating that total hyperfused tissue, not only the necrotic core, has a great impact on EEG alteration. Delta frequency range and an attenuation in the higher part of EEG spectra in stroke are related CBF reduction as an expression of neurovascular coupling.^6^,^22^ Particularly, decrease of rapid frequencies can be observed when CBF drops to 25–35 mL/100 g/min. Marked alterations in brain metabolism occur and regional areas of the cerebral cortex experience failed perfusion when CBF falls below 20–30 mL/100 g/min.^21^ Moreover, considerable decreases of flow (17–18 mL/100 g/min) causes a low frequencies increase, which is associated to progressive neuronal death.^17^ In our hyper-acute study EEG parameters differed highly significantly between early ischemic stroke patients and age matched healthy controls. The observed EEG changes are consistent with findings in sub-acute phase.^13^,^15^,^16^,^37^ Our results showed that higher delta power was associated with higher neurological impairment at admission, while higher alpha power corresponded to extremely low NIHSS values at admission. In turn, this could be explained with the preservation of alpha activity being indicative of neuronal survival in the ischemic regions and good prognosis.^31^ These results are in line with a previous study in which a correlation between early EEG alterations and neurological deficit at admission, as well as a correlation between EEG changes and clinical outcome were observed.^3^ A recent study reported the positive correlation between CTP hypoperfused volume and NIHSS.^19^

EEG alterations, especially in terms of DAR, DTBAR and relative alpha parameters extracted in sub-acute phase were related to functional outcome.^8^,^14^,^15^,^37^ Relative alpha power and delta-to-alpha ratio DAR extracted from sub-acute EEG recordings (49 ± 3 h post symptom onset) were found to be correlated with 30-day NIHSS in study.^15^ Functional outcome in terms of mRS on discharge and at 12 months was associated with delta, alpha, beta relative powers, and DTABR calculated from EEG recorded within 72 h.^8^ A significant correlation between mRS outcome at 6-month and EEG parameters acquired at 3–168 h from stroke onset.^37^

In this study we observed that CTP volumes and EEG spectral parameters were both related to neurologic deficit. A multi-regression stepwise analysis indicated DAR as the strongest parameter related to NIHSS at admission. This finding highlighted the additional informativity of EEG hyper-acute stroke related biomarkers. Quantitative biomarkers may identify systems at risk before overt expression of the disorder. Ideally, biomarkers are sustainable, noninvasive, unaffected to bias and highly available. EEG combines these aspects 27 and provides insight into cortical dysfunction by measuring brain activity directly.^39^ As this pathology entails both neural injury and neural function impairments,^9^,^14^,^25^,^40^ the measure for neural function offered by EEG technique may contribute to decision making in the emergency settings.

The wireless pre-set EEG system allowed hyper-acute bedside EEG assessment in Stroke Unit without compromising regular patient management and delaying treatment. Due to limited space, training and high complexity of the equipment, EEG was considered difficult to perform in emergency departments. Portable wireless EEG devices may overcome the adverse factors making these diagnostic techniques rapidly applicable and easy to use at the bedside in emergency settings.^11^,^12^,^41^ In our study the small-size portable wireless EEG device was placed near to the patient and the acquired analog EEG signals were conditioned and converted to digital signal at a point close to the electrodes, then transmitted *via* Wi-Fi protocol, allowing to reduce of emergency setting environment noise, to minimize electrode wires movement which may be one of the major sources of electromagnetic interference,^42^ as well to limit displacement of the electrodes which may considerably reduce EEG signal quality.

Our study has, however, some limitations. The data describe a single-center investigation of a moderate sample size. EEG recordings were performed at patients’ bedside in the first minutes of hospitalization in an emergency setting. Due to these non-ideal acquisition conditions in stroke unit, artifacts related to the initial medical assessments and nursing care could not be avoided. Thus, it was possible to analyze only relatively short intervals free from artefacts. In our cohort, after EEG recording, patients underwent different types of acute treatment (i.e. thrombolysis alone, thrombolysis and thrombectomy, or no reperfusion treatment). Therefore, due to this inhomogeneity of stroke treatments and their possible different effect on outcomes, reporting consistent data on correlation of EEG with clinical outcome has not been possible. Our study population represents stroke with significant prevalence penumbra compared to ischemic core. However, our study group is a representative sample of anterior circulation stroke, given the median NIHSS score of 8 (3–24) and mean age 78.5 ± 10.9 years.

In conclusion, we assessed the relation between EEG alterations in the earliest phase of ischemic stroke and hypoperfused volumes assessed by CTP, and neurological deficit at admission. We also found that EEG parameters vary depending on the extent of hypoperfused tissue. Moreover, an additional measure of neural function (EEG) and of neural hypoperfusion (CTP) may better depict the impairment level compared to the neuroimaging assessment at admission alone. EEG confirmed to be a sensitive measure for brain function in the earliest phase of cerebral ischemia. These results highlight the added value of EEG as complementary in the evaluation of stroke severity and as a potentially useful tool in monitoring and mapping longitudinal changes in acute stroke patient in the hyper-acute phase.

## References

[1] Adams HPJ, Davis PH, Leira EC, Chang KC, Bendixen BH, Clarke WR, Woolson RF, Hansen MD (1999). Baseline NIH Stroke Scale score strongly predicts outcome after stroke: a report of the Trial of Org 10172 in Acute Stroke Treatment (TOAST). Neurology.

[2] Ajčević M, Furlanis G, Buoite Stella A, Cillotto T, Caruso P, Ridolfi M, Lugnan C, Miladinović A, Ukmar M, Cova MA, Accardo A, Manganotti P, Naccarato M (2020). A CT perfusion based model predicts outcome in wake-up stroke patients treated with recombinant tissue plasminogen activator. Physiol. Meas..

[3] Ajčević M, Furlanis G, Naccarato M, Miladinović A, Buoite Stella A, Caruso P, Cillotto T, Accardo A, Manganotti P (2020). Hyper-acute EEG alterations predict functional and morphological outcomes in thrombolysis-treated ischemic stroke: a wireless EEG study. Med Biol Eng Comput.

[4] Andersen KK, Olsen TS, Dehlendorff C, Kammersgaard LP (2009). Hemorrhagic and ischemic strokes compared: stroke severity, mortality, and risk factors. Stroke.

[5] Assenza G, Zappasodi F, Pasqualetti P, Vernieri F, Tecchio F (2013). A contralesional EEG power increase mediated by interhemispheric disconnection provides negative prognosis in acute stroke. Restor. Neurol. Neurosci..

[6] Astrup J, Siesjö BK, Symon L (1981). Thresholds in cerebral ischemia—the ischemic penumbra. Stroke.

[7] Axel L (1983). Tissue mean transit time from dynamic computed tomography by a simple deconvolution technique. Invest. Radiol..

[8] Bentes C, Peralta AR, Viana P, Martins H, Morgado C, Casimiro C, Franco AC, Fonseca AC, Geraldes R, Canhão P, Pinho e Melo T, Paiva T, Ferro JM (2018). Quantitative EEG and functional outcome following acute ischemic stroke. Clin. Neurophysiol..

[9] Burke Quinlan E, Dodakian L, See J, McKenzie A, Le V, Wojnowicz M, Shahbaba B, Cramer SC (2015). Neural function, injury, and stroke subtype predict treatment gains after stroke. Ann. Neurol..

[10] Chalela JA, Kidwell CS, Nentwich LM, Luby M, Butman JA, Demchuk AM, Hill MD, Patronas N, Latour L, Warach S (2007). Magnetic resonance imaging and computed tomography in emergency assessment of patients with suspected acute stroke: a prospective comparison. Lancet (London, England).

[11] David Hairston W, Whitaker KW, Ries AJ, Vettel JM, Cortney Bradford J, Kerick SE, McDowell K (2014). Usability of four commercially-oriented EEG systems. J. Neural Eng..

[12] Debener S, Minow F, Emkes R, Gandras K, de Vos M (2012). How about taking a low-cost, small, and wireless EEG for a walk?. Psychophysiology.

[13] Finnigan SP, Rose SE, Walsh M, Griffin M, Janke AL, McMahon KL, Gillies R, Strudwick MW, Pettigrew CM, Semple J, Brown J, Brown P, Chalk JB (2004). Correlation of quantitative EEG in acute ischemic stroke with 30-day NIHSS score: comparison with diffusion and perfusion MRI. Stroke.

[14] Finnigan S, van Putten MJAM (2013). EEG in ischaemic stroke: quantitative EEG can uniquely inform (sub-)acute prognoses and clinical management. Clin. Neurophysiol..

[15] Finnigan SP, Walsh M, Rose SE, Chalk JB (2007). Quantitative EEG indices of sub-acute ischaemic stroke correlate with clinical outcomes. Clin. Neurophysiol..

[16] Finnigan S, Wong A, Read S (2016). Defining abnormal slow EEG activity in acute ischaemic stroke: delta/alpha ratio as an optimal QEEG index. Clin. Neurophysiol..

[17] Foreman B, Claassen J (2012). Quantitative EEG for the detection of brain ischemia. Crit. Care.

[18] Furlanis G, Ajcevic M, Buoite Stella A, Cillotto T, Caruso P, Ridolfi M, Cova MA, Naccarato M, Manganotti P (2020). Wake-up stroke thrombolysis reduces ischemic lesion volume and neurological deficit. J. Neurol..

[19] Furlanis G, Ajcevic M, Stragapede L, Lugnan C, Ridolfi M, Caruso P, Naccarato M, Ukmar M, Manganotti P (2018). Ischemic volume and neurological deficit: correlation of computed tomography perfusion with the national institutes of health stroke scale score in acute ischemic stroke. J. Stroke Cerebrovasc. Dis..

[20] Gonzalez RG (2006). Imaging-guided acute ischemic stroke therapy: from “time is brain” to “physiology is brain”. AJNR Am. J. Neuroradiol..

[21] Hertz L (1981). Features of astrocytic function apparently involved in the response of central nervous tissue to ischemia-hypoxia. J. Cereb. Blood Flow Metab..

[22] Hirsch LJ, LaRoche SM, Gaspard N, Gerard E, Svoronos A, Herman ST, Mani R, Arif H, Jette N, Minazad Y, Kerrigan JF, Vespa P, Hantus S, Claassen J, Young GB, So E, Kaplan PW, Nuwer MR, Fountain NB, Drislane FW (2013). American Clinical Neurophysiology Society’s standardized critical care EEG terminology: 2012 version. J. Clin. Neurophysiol..

[23] Jordan KG (2004). Emergency EEG and continuous EEG monitoring in acute ischemic stroke. J. Clin. Neurophysiol..

[24] Kang JM, Cho Y-S, Park S, Lee BH, Sohn BK, Choi CH, Choi J-S, Jeong HY, Cho S-J, Lee J-H, Lee J-Y (2018). Montreal cognitive assessment reflects cognitive reserve. BMC Geriatr..

[25] Kerr AL, Cheng S-Y, Jones TA (2011). Experience-dependent neural plasticity in the adult damaged brain. J. Commun. Disord..

[26] Manganotti P, Furlanis G, Ajčević M, Polverino P, Caruso P, Ridolfi M, Pozzi-Mucelli RA, Cova MA, Naccarato M (2019). CT perfusion and EEG patterns in patients with acute isolated aphasia in seizure-related stroke mimics. Seizure: Eur. J. Epilepsy.

[27] Mikołajewska E, Mikołajewski D (2014). Non-invasive EEG-based brain-computer interfaces in patients with disorders of consciousness. Mil. Med. Res..

[28] Moyanova SG, Dijkhuizen RM (2014). Present status and future challenges of electroencephalography- and magnetic resonance imaging-based monitoring in preclinical models of focal cerebral ischemia. Brain Res. Bull..

[29] Murri L, Gori S, Massetani R, Bonanni E, Marcella F, Milani S (1998). Evaluation of acute ischemic stroke using quantitative EEG: a comparison with conventional EEG and CT scan. Neurophysiol. Clin..

[30] Nagata K, Tagawa K, Hiroi S, Shishido F, Uemura K (1989). Electroencephalographic correlates of blood flow and oxygen metabolism provided by positron emission tomography in patients with cerebral infarction. Electroencephalogr. Clin. Neurophysiol..

[31] Niedermeyer E, Niedermeyer E, Lopes da Silva F (2005). Cerebrovascular disorders and EEG. Electroencephalography: basic principles clinical applications and related fields.

[32] Parsons MW (2008). Perfusion CT: is it clinically useful?. Int. J. Stroke.

[33] Peisker T, Koznar B, Stetkarova I, Widimsky P (2017). Acute stroke therapy: a review. Trends Cardiovasc. Med..

[34] Rossini PM, Altamura C, Ferretti A, Vernieri F, Zappasodi F, Caulo M, Pizzella V, Del Gratta C, Romani G-L, Tecchio F (2004). Does cerebrovascular disease affect the coupling between neuronal activity and local haemodynamics?. Brain.

[35] Schneider AL, Jordan KG (2005). Regional attenuation without delta (RAWOD): a distinctive EEG pattern that can aid in the diagnosis and management of severe acute ischemic stroke. Am. J. Electroneurodiagnostic Technol..

[36] Sharbrough FW, Messick JM, Sundt TM (1973). Correlation of continuous electroencephalograms with cerebral blood flow measurements during carotid endarterectomy. Stroke.

[37] Sheorajpanday RVA, Nagels G, Weeren AJTM, van Putten MJAM, De Deyn PP (2011). Quantitative EEG in ischemic stroke: correlation with functional status after 6 months. Clin. Neurophysiol..

[38] Shreve L, Kaur A, Vo C, Wu J, Cassidy JM, Nguyen A, Zhou RJ, Tran TB, Yang DZ, Medizade AI, Chakravarthy B, Hoonpongsimanont W, Barton E, Yu W, Srinivasan R, Cramer SC (2019). Electroencephalography measures are useful for identifying large acute ischemic stroke in the emergency department. J. Stroke Cerebrovasc. Dis..

[39] Stam C (2006). Nonlinear Brain Dynamics.

[40] Stinear CM, Barber PA, Smale PR, Coxon JP, Fleming MK, Byblow WD (2007). Functional potential in chronic stroke patients depends on corticospinal tract integrity. Brain.

[41] Stragapede L, Furlanis G, Ajcevic M, Ridolfi M, Caruso P, Naccarato M, Ukmar M, Manganotti P (2019). Brain oscillatory activity and CT perfusion in hyper-acute ischemic stroke. J. Clin. Neurosci..

[42] Usakli AB (2010). Improvement of EEG signal acquisition: an electrical aspect for state of the art of front end. Comput. Intell. Neurosci..

[43] Van Kaam RC, van Putten MJAM, Vermeer SE, Hofmeijer J (2018). Contralesional brain activity in acute ischemic stroke. Cerebrovasc. Dis..

[44] Vilela P, Rowley HA (2017). Brain ischemia: CT and MRI techniques in acute ischemic stroke. Eur. J. Radiol..

[45] Welch P (1967). The use of fast Fourier transform for the estimation of power spectra: a method based on time averaging over short, modified periodograms. IEEE Trans. Audio Electroacoust..

[46] Wintermark M, Flanders AE, Velthuis B, Meuli R, van Leeuwen M, Goldsher D, Pineda C, Serena J, van der Schaaf I, Waaijer A, Anderson J, Nesbit G, Gabriely I, Medina V, Quiles A, Pohlman S, Quist M, Schnyder P, Bogousslavsky J, Dillon WP, Pedraza S (2006). Perfusion-CT assessment of infarct core and penumbra: receiver operating characteristic curve analysis in 130 patients suspected of acute hemispheric stroke. Stroke.

[47] Wu J, Srinivasan R, Burke Quinlan E, Solodkin A, Small SL, Cramer SC (2016). Utility of EEG measures of brain function in patients with acute stroke. J. Neurophysiol..

